# Prevalence of Extended Spectrum *β*-Lactamase and Antimicrobial Susceptibility Pattern of Clinical Isolates of* Pseudomonas* from Patients of Khyber Pakhtunkhwa, Pakistan

**DOI:** 10.1155/2016/6068429

**Published:** 2016-06-05

**Authors:** Manzoor Ahmad, Mukhtiar Hassan, Anwar Khalid, Imran Tariq, Muhammad Hassham Hassan Bin Asad, Abdul Samad, Qaisar Mahmood, Ghulam Murtaza

**Affiliations:** ^1^Department of Biochemistry, Faculty of Health Sciences, Hazara University, Mansehra, Khyber Pakhtunkhwa 21120, Pakistan; ^2^Department of Pharmacy, COMSATS Institute of Information Technology, Abbottabad 22060, Pakistan; ^3^University College of Pharmacy, University of Punjab, Lahore 54000, Pakistan; ^4^Department of Environmental Sciences, COMSATS Institute of Information Technology, Abbottabad 22060, Pakistan

## Abstract

Majority of gram negative pathogenic bacteria are responsible for extended spectrum *β*-lactamases (ESBLs) production, which show resistance to some newer generation of antibiotics. The study was aimed at evaluating the prevalence of ESBL and antibiotic susceptibility pattern of* Pseudomonas* isolates collected during 2010 to 2014 from tertiary care hospitals of Peshawar, Pakistan. Out of 3450 samples, 334* Pseudomonas* spp. isolates comprised of 232 indoor and 102 outdoor patients were obtained from different specimens and their susceptibility pattern was determined against 20 antibiotics. Antimicrobial susceptibility testing was carried out using the Kirby-Bauer agar diffusion method and ESBL production was detected by Synergy Disc Diffusion technique. The mean age group of the patients was 29.9 + 9.15 years. Meronem showed best activity (91.02%) from class carbapenem, *β*-lactam and *β*-lactamase inhibitors exhibited 69.16% activity, and doxycycline had a diminished activity (10.18%) to* Pseudomonas* spp. Outdoor isolates were more resistant than the indoor and during the course of the study the sensitivity rate of antibiotics was gradually reducing. ESBL production was observed in 44.32% while the remaining was non-ESBL. The moderate active antibiotics were amikacin (50.7%), SCF (51.4%), TZP (52.7%), and MXF (54.1%) among ESBL producing isolates. Lack of antibiotic policy, irrational uses (3GCs particularly), and the emergence of antibiotic resistant organisms in hospitals may be causes of high antibiotic resistance.

## 1. Introduction

Genus* Pseudomonas* is an important member of the family Pseudomonadaceae. They are in a straight or sometimes in marginally bent form in shape, characteristically aerobic in nature, and flagellated [1]. According to Obritsch et al. [2],* Pseudomonas* is in the third rank to cause UTIs and dermatitis, otitis, conjunctivitis, GIT, soft tissue, bone, and joint infections are also often caused by these species [3]. Studies conducted on HIV-infected patients reported a progressive increase of gram-negative bacilli, including* Pseudomonas *spp. In fact,* Pseudomonas *spp. induced invasive infection is observed with growing frequency among immunocompromised hosts and patients with some predisposing conditions, such as malignancies, extremes of age, neutropenia, prolonged hospitalization, surgery, trauma, and instrumentation [4].

*Pseudomonas* strains were isolated from wound of burn patients (22 to 73%) [5]. It is the main causative agent of morbidity and mortality in patients of grown age with cystic fibrosis of the respiratory tract infection [6]. The antimicrobial resistance is one of the most important complications which are associated with* Pseudomonas*. A limited number of antibiotics are effective, because of their tendency to be resistant naturally to a number of pathogens [7], and resistance rises at an elevated frequency to those therapeutic drugs that were previously sensitive to* Pseudomonas *[8]. Antimicrobial resistance in nosocomial settings is linked with adverse clinical consequences and higher costs [9]. Extended spectrum beta lactamases (ESBLs) are the enzymes that hydrolyze and induce resistance to cephalosporin monobactams [10]. Various studies from subcontinent suggested the prevalence rate of ESBL production of* Pseudomonas *spp. is 22 to 36% [11, 12]. The two drugs' synergetic activity is considered to be most effective in the treatment of pseudomonal infections, by using penicillin in combination with aminoglycosides and carbapenems (*β*-lactamase inhibitors) or antipseudomonal penicillin alone [13].

The purpose of this study is to determine the rate of occurrence, pattern of antimicrobial susceptibility, and prevalence of ESBL production of these bacteria from three main tertiary care hospitals in Peshawar region of KPK, Pakistan, as limited work has been previously conducted on this subject.

## 2. Methodology

### 2.1. Collection of Bacterial Isolates (Specimens)

This study was conducted in Microbiology Section at Pathology Department, Khyber Teaching Hospital, Peshawar, from 2010 to 2014. During the study period, 3450 samples were collected from three main tertiary care hospitals of Peshawar KPK, Pakistan. The specimens collected were comprised of pus (wounds, burns, ear, TS, and HVS), urine, and blood from indoor patients of gynaecology, surgery, medicine, and burns units and outdoor patients.

The samples from the suspected patient were introduced to inoculation on blood agar, nutrient agar, MacConkey agar, and CLED agar and, after overnight aerobic incubation at 37°C, they were examined for bacterial growth. Grown colonies were isolated and identified on the basis of their colony morphology, staining characters, pigment production, motility, and relevant biochemical tests as per standard laboratory methods of identification [14, 15].

### 2.2. Antimicrobial Susceptibility Protocol

For inoculums preparation, tryptic soy broth (CM129-OXOID) was made by pouring 4-5 mL of broth medium in screw capped tubes and sterilized by autoclaving at 121°C for 15 minutes at 15 psi. The media were cooled and kept in an incubator for 24 hours at 35°C prior to inoculation. The inoculum density was standardized to a final concentration of 1.5 × 10^8^ CFU/mL according to CLSI and placed in an incubator for 2–6 hours at 35°C to check sterility.

Routinely used different groups of antibiotics (purchased from Oxoid, England) were subjected to determine the antimicrobial susceptibility pattern by using disc diffusion method [16, 17]. Minimum inhibitory concentrations (MICs) were found by agar dilution method for representative antibiotics of different groups. The standard break points were standardized with CLSI reported by Cockerill [18].

### 2.3. Detection of ESBL

The isolates were screened according to CLSI as prescribed by Hawser et al. [19], to assess the prevalence of ESBL in* Pseudomonas *spp. Isolates stored at −20°C were refreshed on tryptic soy agar medium for ESBL production by using disc diffusion method.

### 2.4. Synergy Disc Diffusion Method

In the initial screening of ESBL production, disc diffusion method was used. Discs of cefotaxime (CTX = 30 *μ*g), ceftazidime (CAZ = 30 *μ*g), ceftriaxone (CRO = 30 *μ*g), and aztreonam (AZM = 30 *μ*g) were placed at a distance of 25–30 mm from amoxicillin + clavulanic acid. Amoxicillin + clavulanic acid (AMC = 20 + 10 *μ*g) was placed in the center of the inoculated plates containing Müller Hinton agar according to the CLSI recommendations [19]. Zones of inhibition around the 3G cephalosporin discs and aztreonam were observed after overnight incubation at 37°C. When the zones of inhibition around the third-generation cephalosporin discs and aztreonam were extended on the sides nearest to AMC, the isolate is said to be ESBL producer.

### 2.5. Phenotypic Detection of ESBL

In the phenotypic confirmatory test, the test organisms were inoculated on Müller Hinton agar (MHA) and discs of ceftazidime (30 *μ*g) and cefotaxime (30 *μ*g) alone and a disc in combination with clavulanic acid (30/10 *μ*g) were placed on the inoculated agar for each isolate. Both the discs were placed 25 mm apart (center to center) on a lawn culture of the test plate and incubated for 24 hours at 37°C. An increase in zone of (≥5 mm) for either antimicrobial agent tested in combination with clavulanic acid versus its zone when tested alone was designated as ESBL positive.* Klebsiella pneumonia *ATCC (700603) and* E. coli *ATCC (25922) were used as positive and negative control strains, respectively.

### 2.6. Statistical Analysis

The data were analyzed by using *X* ^2^ test through SPSS version 16.0.

## 3. Results

**Figure 1:**
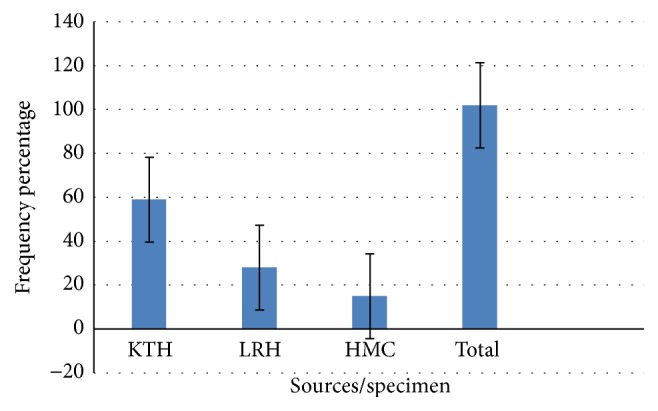
Prevalence rate of* Pseudomonas *spp. isolates from different specimen.

A total of 3450 samples were collected from the three main tertiary care hospitals of Peshawar, namely, Khyber Teaching Hospital, Lady Reading Hospital, and Hayatabad Medical Complex.* Pseudomonas *spp. obtained from different sources are listed in Figure 1. These samples were further screened for antimicrobial susceptibility and ESBLs prevalence. Specimens were taken from different sources: pus 162 (48.50%), urine 67 (20.05), blood 16 (4.79%), and HVS, along with throat and ear swabs 32 (9.58%).

**Figure 2:**
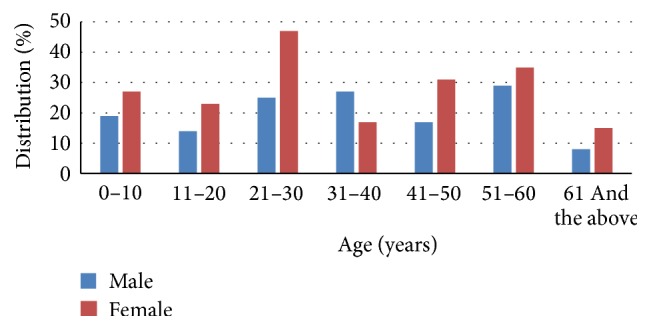
Genderwise distribution of male and female among different age groups.

**Figure 3:**
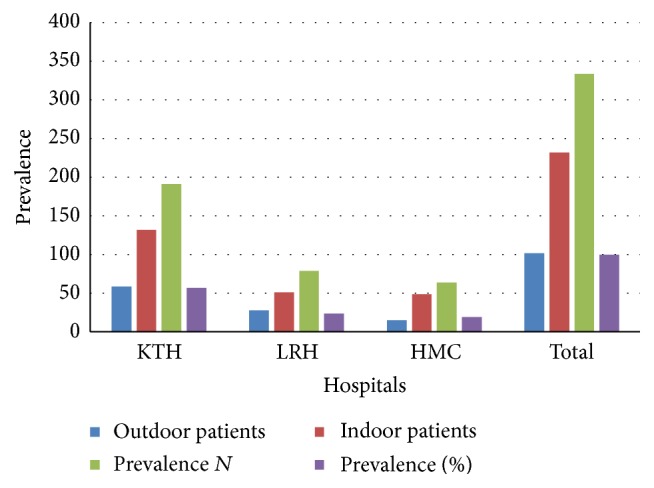
Prevalence of* Pseudomonas* spp. in different hospitals.

A total of 334 isolates comprising 232 (69.46%) indoor and 102 (30.54%) outdoor patients including males and females as mentioned in Figure 2 (male to female ratio 1 : 1.4) were recovered as* Pseudomonas* positive, out of which most of the isolates were taken from Khyber Teaching Hospital 191 (57.18%), followed by Lady Reading Hospital 79 (23.65%) and Hayatabad Medical Complex, Peshawar 64 (19.16%) with mean age 25.9 ± 9.15 years. Details are given in Figure 3.

### 3.1. Susceptibility Pattern of* Pseudomonas *spp. to Various Antimicrobial Agents

In *β*-lactam agents, the frequency of susceptibility to cephalosporins' 2nd generation (cefaclor) 21.26%, 3rd generation (ceftazidime and ceftriaxone) 33.23% and 36.23%, respectively, while the 4th generation (cefepime) was 48.5%, showed a higher activity among the cephalosporin's group. While, in *β*-lactam agents, the most potent antimicrobial agent was imipenem (84.43%) and Meronem (91.02%) from class carbapenems against* Pseudomonas *spp., least activity was observed by amoxicillin 14.97%.

Among the *β*-lactams and *β*-lactamase inhibitors, maximum activity was observed by cefoperazone + sulbactam (69.16%) and then by piperacillin + tazobactam (60.78%) and amoxicillin + clavulanic acid (24.5%). Susceptibility pattern against amikacin was 66.0% and gentamycin was 19.6% in aminoglycosides.

In class fluoroquinolones, 61.7% isolates were found susceptible to moxifloxacin, 44.6% to gatifloxacin and 55.7% to sparfloxacin, 50.3% ciprofloxacin, and 35% to enoxacin. In the macrolides group of antibiotics, clarithromycin had a good activity (42.51%), while to erythromycin it was 19.16%. In class tetracycline, 10.18% strains were found sensitive to doxycycline. Nevertheless, none of the antibiotics was found completely resistant to the* Pseudomonas *spp. The resistance rate was highest for tetracycline followed by penicillin and the isolates were coresistant to macrolides and fluoroquinolones (Table 1).

### 3.2. Susceptibility Pattern of Strain in Indoor and Outdoor Patients

Among the 334* Pseudomonas* positive isolates, 102 were from outdoor patients while 232 were recovered from indoor patients. Outdoor isolates showed a higher frequency of sensitivity to almost all the antibiotics (Table 2). Imipenem (88.79%) and Meronem (96.08%) were highly active antibiotics from the class of carbapenems in indoor isolates, while outdoor isolates were 81.47% and 91.18% susceptible towards these two antibiotics. 30.39% isolates were susceptible to cefaclor, 41.18% to ceftazidime, 44% to ceftriaxone, and 59% to cefepime (4th generation cephalosporin) of *β*-lactam agents from outdoor isolates, while 17.2% were susceptible to cefaclor, 29.24% to ceftazidime, 32.76% to ceftriaxone, and 44% to cefepime for indoor patients. These results showed that the frequency of susceptibility is higher for outdoor isolates.

Amongst the *β*-lactams and *β*-lactamase (combined) inhibitors, cefoperazone-sulbactam was 65% active, piperacillin-tazobactam was 56.9%, and Augmentin was 19.4% active against indoor isolates of* Pseudomonas* spp., while the percent activities of these combined antibiotics against the outdoor isolates of the pathogen were 78.43%, 69.6%, and 36.27% by cefoperazone-sulbactam, piperacillin-tazobactam, and amoxicillin + clavulanic, respectively. Amoxicillin showed 9.48% (indoor) and 27.45 (outdoor) susceptibility rates to the positive isolates.

Moxifloxacin had a maximum activity among the fluoroquinolones against outdoor isolated followed by ciprofloxacin, sparfloxacin, gatifloxacin, and then enoxacin with 70.59%, 58.82%, 56.86%, 52.94%, and 41.18% sensitivity, respectively. In contrast, the rate of susceptibility of moxifloxacin was 57.76%, sparfloxacin 55.17%, ciprofloxacin 46.55%, gatifloxacin 40.95%, and enoxacin 32.33% in hospitalized patients.

In amino glycosides, amikacin (indoor patients = 60.34%, outdoor patients = 74.51%) had a better activity than gentamycin (16.38 and 23.53% indoor and outdoor patients, resp.). In doxycycline, the only tetracycline that had a diminished rate of activeness for both indoor and outdoor patients, 5.17% and 21.57% (Table 3). Overall susceptibility rate in the hospital was affected which might be due to increase of use of antibiotics and nosocomial environment.

### 3.3. Yearwise Susceptibility Pattern of Isolates to Different Antibiotics

The changes in the susceptibility pattern during the study period from 2010 to 2014 against all types of clinical specimens were checked for various classes of antibiotics. The sensitivity pattern of combined *β*-lactams and *β*-lactamase inhibitors was 77.8% in 2010 and 70.5% in 2011, while a slight drift has been seen in the last 3 consecutive years which was 66%, 68%, and 61% for 2012-2013 and 2014 against SCF. AMC showed an average 15% sensitivity rate throughout the study period. The range was acceptable that is 11.9%–18.5%.

Carbapenem exhibited greater activity over other antimicrobial agents against* Pseudomonas *spp. during the course of the study. Within the same class, Meronem had a better activity than the imipenem over the entire duration; however, both carbapenems had a range of 92.8% to 89.4% and 85.3% to 92%, respectively.

Among the beta lactams, all generation of cephalosporin used in the study had consistently diminished rate of sensitivity: cefaclor was 24.1% sensitive in 2010, while it has fluctuated to 19% in 2014; ceftazidime had an average of 34.14% for the entire period. However, the 4th generation cephalosporin had shown an increase in effectiveness rate from 53.7% in 2010 to 56.4% in 2011 and then a sudden decline (43%) in the next three years.

Relatively a steady decrease has been examined with the following percent of susceptibility rates to SPX over the study periods 63.8%, 56.4% 54.3%, 53%, and then 52.4%. Among the fluoroquinolones, sensitivity rate for GTX was found 52% and 50% in 2010 and 2011, respectively. However, a gradual decrease was noted in the next three years (42.6%, 40.9%, and 35.7%). CIP frequency range was 45.2% to 57.4%. Also for MXF, isolates showed a reduction in susceptibility ranges from 68.5% in 2010 to 57.1% in 2014.

Erythromycin and clarithromycin were the least reliably active reagents against the tested* Pseudomonas *spp. Overall, there was a moderate decrease in susceptibility rate to the antibiotic analyzed over the last five years of the study. Table 3 reflects yearwise susceptibility pattern of isolates against individual antibiotic.

### 3.4. MIC (Minimum Inhibitory Concentrations)

Percent susceptible strains and MICs 50% and 90% inhibition range were calculated for each of the antibiotics. The values show concordance between disc zone diameter (mm) and MICs values for antibiotics (Table 4).

**Figure 4:**
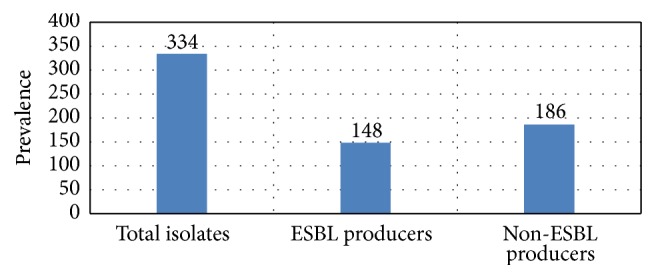
Prevalence of ESBL in clinically pathogenic* Pseudomonas *spp.

Production of ESBLs was observed in 148 (44.32%) of the isolates and the remaining 186 (55.68%) were non-ESBL producers (Figure 4). A high resistance rate was observed in the ESBL positive isolates as compared to non-ESBL strains (Table 5).

A significant difference was found in susceptibility to the carbapenems, quinolones, and *β*-lactam/*β*-lactamase inhibitors, statistically. Resistance of ESBLs to the other class of antibiotics like penicillin and macrolides was a little a bit higher than the non-ESBLs but statistically it was not significant.

The resistance conferred by ESBLs producing* Pseudomonas *spp. to cephalosporin (CEC, CAZ, CRO, and CFP) was 14.2%, 20.3%, 14.3%, and 22.3%, respectively, contrary to the non-ESBLs. Both ESBL and non-ESBL producers isolates were almost resistant to tetracycline. Good susceptibility was observed with amikacin in both ESBL (50.7%) and non-ESBL producers (75.8%). On the other hand, pseudomonal susceptibility against antibiotics from the *β*-lactams and *β*-lactamase inhibitors was observed. Augmentin was 16.2% and 31.2% for ESBL and non-ESBL producers, respectively. It was 37.2% and 50.5% for ESBLs and non-ESBLs producing isolate against GTX. Noble activity has been shown for both ESBL and non-ESBL by the class carbapenems and the considerable activities by SCF, MXF, and AK, which has a higher activity than cephalosporin. A higher resistance for AMC, AML, and DO was evaluated in comparison to SPR and SPX of *β*-lactam inhibitors and CLR and CIP member of quinolones given in Table 5.

### 3.5. ESBL and Non-ESBL Producing* Pseudomonas *spp. (Hospitalwise)

**Figure 5:**
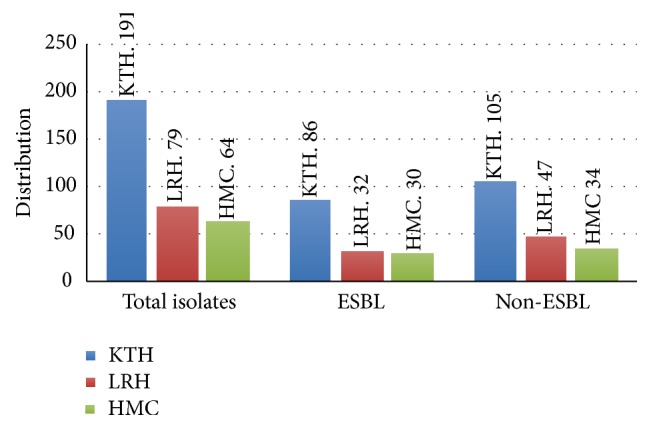
Hospitalwise distribution of ESBL and Non-ESBL producing* Pseudomonas *spp.

The overall frequency of isolation of ESBL producing organism at different hospitals is depicted in Figure 5. The distribution of ESBL isolates varied, considerably in each hospital. The highest incidence (46.88%) was observed at Hayatabad Medical Complex (HMC) followed (45.03%) by Khyber Teaching Hospital (KTH). The prevalence was comparatively small (40.51%) from Lady Reading Hospital (LRH).

The statistical analysis indicates that there is no association between ESBL and hospitals; means hospitals are not involved to produce ESBL. While in outdoor patients females are 1.158 times more likely than male patients who produce positive ESBL, male indoor patients are 0.802 times less likely than female outdoor patients who produce positive ESBL

## 4. Discussion

Surveillance is a key to the control of antimicrobial resistance. Facts and figures found by surveillance events can be used to direct empirical prescribing of antimicrobial agents, to identify newly developing resistances, to determine importance for research, and to evaluate involvement strategies and potential control trials aimed at dropping the prevalence of resistant pathogens [20].

The rate of susceptibility was most productive for antimicrobial agent of class carbapenem against* Pseudomonas *spp. [21]. Supported current results as 90% of strains were susceptible to Meronem and 84% to imipenem of class carbapenems.

The results are in agreement with Zhanel et al.'s [22] findings as reported for both sparfloxacin and moxifloxacin 58%, followed by ciprofloxacin 46.7%. In this study, amoxicillin and amoxiclav have established 85% and 78.44% resistance. The study conducted in Pakistan reported by Khan et al. [23] had a high resistance rate of penicillin that is 98%; our findings are also in agreement with other studies as reported by Sasirekha et al. [24] and Ullah et al. [12] with respect to penicillin's. In the present study cefaclor, ceftriaxone, ceftazidime, and cefepime were found to be 17.24%, 33.19%, 33.62%, and 43.97% acquired from hospital and 30.39%, 35.29%, 44.12%, and 58.82% susceptible from the outdoor (OPD) patients infected by* Pseudomonas* spp., respectively. Susceptibility to fourth-generation cefepime reported in India was 32% [25] and in Bulgaria 42% [26] against* Pseudomonas *spp. isolates and cephalosporin especially third generation has been used for gram-negative bacteria treatment [27]. The results of the third generation are in close agreement with other studies [26, 28].

Aminoglycosides have good activity against clinically important gram-negative bacilli [29]. In the present study among the non-beta lactams, 68.53% isolates were susceptible to amikacin, followed by 16.38% to gentamicin. It was similar to what is reported in the literature [24]; in France a higher susceptibility rate of 86% of amikacin was reported by Cavallo et al. [30]. Several studies showed that amikacin was more sensitive than gentamicin and our results also support the above arguments. In 2010 gentamicin was 59% resistant in India [24] and 55.5% in Bangladesh [31], while in Bulgaria it was 36.11% recorded in* Pseudomonas *spp. by Strateva et al. [26].

Pseudomonads have more adoptability than Enterobacteriaceae in developing drug resistance by diverse means. The production of ESBLs found more resistance at different stages to expanded spectrum cephalosporins [32].

Production of *β*-lactamases by bacteria which hydrolyzes the penicillin, cephalosporin, and other *β*-lactams leads to inactivation of the drug. Extended spectrum beta lactamases (ESBLs) are enzymes that hydrolyze and induce resistance to cephalosporins (ceftriaxone, ceftazidime, cefepime, and cefotaxime) and monobactams (aztreonam) [10, 33]. Cross resistance in* Pseudomonas *spp. has been identified to be the emerging risk factor to imipenem, formerly used fluoroquinolone [34], and a study reported that imipenem-resistant to * Pseudomonas *spp. isolates showed resistance to ciprofloxacin or levofloxacin, signifying that cross resistance is established for imipenem [35]. Multidrug resistant* Pseudomonas* isolates, sometimes, develop Pan-drug resistance, resistant to all the antibiotics except colistin [35]. *β*-lactams are hydrophilic antibiotics, utilizing the pore forming proteins (OprD in* Pseudomonas and *OmpF in* E. coli*) to enter inside the cell, whereas larger hydrophobic antibiotics and macrolides cross the lipid bilayer through diffusion. Alteration in lipid or proteins conformation of OM barrier accounts for survival of the antibiotic-resistant strains and pinpoints its position in antibiotic susceptibility [36].

ESBLs prevalence in this particular study was recorded as 44.32%, which was very similar to the studies conducted by (Ali et al. [37], Jabeen et al. [38], and Ullah et al. [12]) from Pakistan, 40%, 43%, and 58.7% ESBLs producers. Lower rates were recorded by Anjum and Mir [39] in Pakistan which observed 33% and this incidence is superior to continental surveys performed in South America (18.1%), Europe (11%), North America (7.5%), and Asia-Pacific (14.2%) parts [19, 40].

The findings of this study indicate that indoor ESBLs producing* Pseudomonas* isolates were more resistant towards third-generation antibiotics, for example, cephalosporins (78% to 86%) as compared with outdoor isolates, supported by the studies of Babypadmini and Appalaraju [41], who found 84%, and Sasirekha et al. [24], who reported 75 to 85% resistance rate to cephalosporins. Most of the ESBLs producing organisms were found coresistant to fluoroquinolones and aminoglycosides which correlate with the study done by Denholm et al. [42] and Jabeen et al. [38]. It is because of the genes, encoding that these *β*-lactamases are often situated at large plasmids which also encode resistant genes for other antibiotics, together with sulfonamides, tetracycline, chloramphenicol, trimethoprim, and aminoglycosides [43].

Use of cephalosporin is not only associated with ESBL infection, but also seen to be a risk factor for colonization with ESBL producing organisms [44]. As a result, higher percentage of ESBL producing* Pseudomonas *spp. because of selected stress is forced by excessive use of the 3rd-generation cephalosporins in this research. This association has been best displayed by interventional study which demonstrated decline in the frequency of ESBL pooling from 8% to 6% due to control use of third-generation cephalosporins [45]. ESBLs occurrence was significantly higher among isolates from inpatients than outpatients (*P* = 0.002).

In this study, we used two combinations with clavulanic acid (CAZ/CAZC and CTX/CTXC) and found that* Pseudomonas *spp. revealed higher production of ESBLs in CAZ/CAZC and are closely proximate to the findings of other researches [46, 47]. As a result, laboratories should perform the ESBLs confirmatory test to both resistant and sensitive strains. The marker of ESBLs that is cefotaxime, ceftazidime, and ceftriaxone is no longer recommended.

Consequently a quick response is needed to identify the ESBLs producing organisms that in future proper antibiotic practice and infection managing procedures can be employed.

## 5. Conclusion

High level of resistance of antibiotics to* Pseudomonas *spp. may be due to lack of antibiotic policy, irrational use of antibiotics, particularly 3GCs, and the emergence of antibiotic-resistant organisms in hospitals, resulting in multidrug resistance in* Pseudomonas* spp.

## Figures and Tables

**Table 1:** Cumulative susceptibility pattern of *Pseudomonas *spp. to various antimicrobial agents.

Antimicrobial agent	Codes	Sensitive *N* (%)	Intermediate *N* (%)	Resistant *N* (%)
Amoxicillin	AML	50 (14.97)	39 (11.68)	245 (73.35)
Amoxicillin + clavulanic acid	AMC	82 (24.55)	26 (7.78)	226 (67.66)
Piperacillin + tazobactam	TZP	203 (60.78)	55 (16.47)	76 (22.75)
Cefoperazone + sulbactam	SCF	231 (69.16)	44 (13.17)	59 (17.66)
Cefaclor	CEC	71 (21.26)	22 (6.59)	241 (72.16)
Ceftazidime	CAZ	111 (33.23)	27 (8.08)	196 (58.68)
Ceftriaxone	CRO	121 (36.23)	37 (11.08)	176 (52.69)
Cefepime	FEP	162 (48.50)	17 (5.09)	155 (46.41)
Sparfloxacin	SPX	186 (55.69)	14 (4.19)	134 (40.12)
Ciprofloxacin	CIP	168 (50.30)	19 (5.69)	147 (44.01)
Gatifloxacin	GTX	149 (44.61)	84 (25.15)	101 (30.24)
Enoxacin	ENX	117 (35.03)	37 (11.08)	180 (53.89)
Moxifloxacin	MXF	206 (61.68)	35 (10.48)	93 (27.84)
Erythromycin	E	64 (19.16)	22 (6.59)	248 (74.25)
Clarithromycin	CLR	142 (42.51)	37 (11.08)	155 (46.41)
Meropenem	MEM	304 (91.02)	4 (1.20)	26 (7.78)
Imipenem	IPM	282 (84.43)	15 (4.49)	37 (11.08)
Gentamycin	CN	62 (18.56)	24 (7.19)	248 (74.25)
Amikacin	AK	216 (64.67)	17 (5.09)	101 (30.24)
Doxycycline	DO	34 (10.18)	19 (5.69)	281 (84.13)

**Table 2:** Comparative susceptibility pattern between indoor and outdoor patients.

Antimicrobial agents	Hospitalized patients *n* = 232		Outdoor patients *n* = 102		*P* value
	Sensitive *N* (%)	Resistant *N* (%)	Sensitive *N* (%)	Resistant *N* (%)	
AML	22 (9.48)	210 (90.52)	28 (27.45)	74 (72.55)	<0.001
AMC	45 (19.40)	187 (80.60)	37 (36.27)	65 (63.73)	<0.0001
TZP	132 (56.90)	100 (43.10)	71 (69.61)	31 (30.39)	<0.03
SCF	151 (65.09)	81 (34.91)	80 (78.43)	22 (21.57)	<0.02
CEC	40 (17.24)	192 (82.76)	31 (30.39)	71 (69.61)	<0.006
CAZ	69 (29.74)	163 (70.26)	42 (41.18)	60 (58.82)	<0.041
CRO	76 (32.76)	156 (67.24)	45 (44.12)	57 (55.88)	<0.467
FEP	102 (43.97)	130 (56.03)	60 (58.82)	42 (41.18)	<0.0123
ENX	75 (32.33)	157 (67.67)	42 (41.18)	60 (58.82)	<0.022
GTX	95 (40.95)	137 (59.05)	54 (52.94)	48 (47.06)	<0.05
CIP	108 (46.55)	124 (53.45)	60 (58.82)	42 (41.18)	<0.0388
SPX	128 (55.17)	104 (44.83)	58 (56.86)	44 (43.14)	<0.0427
MXF	134 (57.76)	98 (42.24)	72 (70.59)	30 (29.41)	<0.03
E	35 (15.09)	197 (84.91)	29 (28.43)	73 (71.57)	<0.0137
CLR	88 (37.93)	144 (62.07)	54 (52.94)	48 (47.06)	<0.0106
CN	38 (16.38)	194 (83.62)	24 (23.53)	78 (76.47)	<0.0193
AK	140 (60.34)	92 (39.66)	76 (74.51)	26 (25.49)	<0.01
DO	12 (5.17)	220 (94.83)	22 (21.57)	80 (78.43)	<0.0001
MEM	206 (88.79)	26 (11.21)	98 (96.08)	4 (3.92)	<0.032
IPM	189 (81.47)	43 (18.53)	93 (91.18)	9 (8.82)	<0.0242

**Table 3:** Yearwise susceptibility pattern (sensitivity) of *Pseudomonas* spp. to different antibiotics.

Agents	2010 *N* (%)	2011 *N* (%)	2012 *N* (%)	2013 *N* (%)	2014 *N* (%)	Total *N* (%)
AML	10 (18.5)	14 (17.9)	13 (13.8)	8 (12.1)	5 (11.9)	50 (15.0)
AMC	17 (31.5)	22 (28.2)	18 (19.1)	17 (25.8)	8 (19.0)	82 (24.6)
TZP	35 (64.8)	47 (60.3)	57 (60.6)	40 (60.6)	24 (57.1)	203 (60.8)
SCF	42 (77.8)	55 (70.5)	63 (67.0)	45 (68.2)	26 (61.9)	231 (69.2)
CEC	13 (24.1)	17 (21.8)	19 (20.2)	14 (21.2)	8 (19.0)	71 (21.3)
CAZ	22 (40.7)	27 (34.6)	29 (30.9)	23 (34.8)	10 (23.8)	111 (33.2)
CRO	21 (38.9)	30 (38.5)	33 (35.1)	20 (30.3)	17 (40.5)	121 (36.2)
FEP	29 (53.7)	44 (56.4)	41 (43.6)	29 (43.9)	19 (45.2)	162 (48.5)
SPX	34 (63.0)	44 (56.4)	51 (54.3)	35 (53.0)	22 (52.4)	186 (55.7)
CIP	31 (57.4)	37 (47.4)	47 (50.0)	34 (51.5)	19 (45.2)	168 (50.3)
ENX	21 (38.9)	25 (32.1)	34 (36.2)	24 (36.4)	13 (31.0)	117 (35.0)
GTX	28 (51.9)	39 (50.0)	40 (42.6)	27 (40.9)	15 (35.7)	149 (44.6)
MXF	37 (68.5)	52 (66.7)	55 (58.5)	38 (57.6)	24 (57.1)	206 (61.7)
E	11 (20.4)	14 (17.9)	18 (19.1)	13 (19.7)	8 (19.0)	64 (19.2)
CLR	27 (50.0)	37 (47.4)	38 (40.4)	24 (36.4)	16 (38.1)	142 (42.5)
MEM	49 (90.7)	72 (92.3)	85 (90.4)	60 (90.9)	38 (90.5)	304 (91.0)
IPM	46 (85.2)	67 (85.9)	79 (84.0)	54 (81.8)	36 (85.7)	282 (84.4)
CN	11 (20.4)	15 (19.2)	18 (19.1)	11 (16.7)	7 (16.7)	62 (18.6)
AK	36 (66.7)	49 (62.8)	63 (67.0)	42 (63.6)	26 (61.9)	216 (64.7)
DO	7 (13.0)	9 (11.5)	9 (9.6)	6 (9.1)	3 (7.1)	34 (10.2)

**Table 4:** In vitro susceptibility of ESBL and non-ESBL producing *Pseudomonas *spp. shows the MICs against the tested strain (MIC 50 *μ*g/mL and MIC 90 *μ*g/mL).

Antibiotics	% susceptibility	MIC 50 *μ*g/mL	MIC 90 *μ*g/mL
AMC	21.56	16	32
AML	14.97	32	64
SCF	66.17	4	16
CEC	21.26	128	256
CAZ	34.13	64	64
CRO	36	32	128
FEP	48.5	8	32
CIP	46.71	2	16
GTX	52.40	16	128
MXF	58.08	0.5	2
MEM	91.02	1	4
IPM	87.43	1	8
CN	19.16	16	32
AK	67.07	4	4
DO	10.18	256	512

**Table 5:** In vitro (%) susceptibility of ESBL and non-ESBL produced by *Pseudomonas *spp.

Antimicrobials	ESBL producers		Non-ESBL producers		*P* value
	Susceptible *N* (%)	Resistant *N* (%)	Susceptible *N* (%)	Resistant *N* (%)	
AML	14 (9.5)	134 (90.5)	36 (19.4)	150 (80.6)	<0.018
AMC	24 (16.2)	124 (83.8)	58 (31.2)	128 (68.8)	0.0025
TZP	78 (52.7)	70 (47.3)	125 (67.2)	61 (32.8)	<0.01
SCF	76 (51.4)	72 (48.6)	155 (83.3)	31 (16.7)	<0.0001
CEC	21 (14.2)	127 (85.8)	50 (26.9)	136 (73.1)	<0.007
CAZ	30 (20.3)	118 (79.7)	81 (43.5)	105 (56.5)	<0.0001
CRO	22 (14.3)	126 (85.1)	99 (53.2)	87 (46.8)	<0.0001
FEP	33 (22.3)	115 (77.7)	129 (69.4)	57 (30.6)	<0.0001
SPX	62 (41.9)	86 (58.1)	124 (66.7)	62 (33.3)	<0.0001
CIP	55 (37.2)	93 (62.8)	113 (60.8)	73 (39.2)	<0.0001
ENX	43 (29.1)	105 (70.9)	74 (39.8)	112 (60.2)	<0.05
GTX	55 (37.2)	93 (62.8)	94 (50.5)	92 (49.5)	<0.01
MXF	80 (54.1)	68 (45.9)	116 (62.4)	70 (37.6)	<0.13
E	36 (24.3)	112 (75.7)	28 (15.1)	158 (84.9)	<0.325
CLR	66 (44.6)	80 (54.1)	76 (40.9)	110 (59.1)	<0.43
MEM	128 (86.5)	20 (13.5)	176 (94.6)	10 (5.4)	<0.0098
IPM	120 (81.1)	28 (18.9)	172 (92.5)	14 (7.5)	<0.0018
CN	20 (13.5)	128 (86.5)	42 (22.6)	144 (77.4)	<0.034
AK	75 (50.7)	73 (49.3)	141 (75.8)	45 (24.2)	<0.0001
DO	9 (6.1)	139 (93.9)	25 (13.4)	161 (86.6)	<0.0426
